# Une cataracte de dessiccation en “plumes de paon”

**DOI:** 10.11604/pamj.2018.31.68.16235

**Published:** 2018-10-01

**Authors:** Ghita Bouayad, Abdelbarre Oubaaz

**Affiliations:** 1 Université Mohammed V Souissi, Service d’Ophtalmologie de l’Hôpital Militaire d’Instruction Mohamed V, Hay Riad, Rabat, Maroc

**Keywords:** Cataracte, dessiccation, plumes, Cataract, desiccation, feathers

## Abstract

Desiccation cataract is a common complication of posterior vitrectomy. Indeed, the contact between internal gas tamponade and posterior crystalloid can cause early posterior subcapsular cataract occurring in the twenty-four hours, also known as fern leaf-like cataract. It is caused by dehydration of the posterior layers of the crystalline lens or by a block in the metabolic exchanges from both sides of the posterior crystalloid. Histological examination shows vacuoles or feather-like linear opacities in the posterior subcapsular cortex, in most cases regressive. However, long-acting gases induce permanent clouding of cristalline lens, which is often very adherent and whose dissection during phakoexeresis may be complicated by posterior capsular rupture. We report the case of a 54 year old patient undergoing vitrectomy for retinal detachment in the right eye associated with cryopexy of the retinal dehiscence and sulfur hexafluoride (SF6) tamponade. One day postoperatively examination showed peacock feathers-like posterior subcapsular cataract.

## Image en médecine

La cataracte de dessiccation est une complication fréquente de la chirurgie du segment postérieur par vitrectomie. En effet, le contact entre le gaz utilisé pour le tamponnement interne et la cristalloïde postérieure peut induire une cataracte précoce sous-capsulaire postérieure, survenant dans les vingt-quatre heures, aussi dénommée en «feuille de fougère». Celle-ci est liée à une déshydratation des couches postérieures du cristallin ou à un blocage des échanges métaboliques de part et d'autre de la cristalloïde postérieure. Histologiquement, on retrouve des vacuoles ou des opacités linéaires en forme de plume au niveau du cortex sous-capsulaire postérieur, le plus souvent régressives. Cependant, les gaz de longue durée d'action favorisent une opacification permanente du cristallin, qui est souvent très adhérente et dont la dissection lors d'une phakoexérèse peut se compliquer d'une rupture capsulaire postérieure. Nous rapportons le cas d'un patient âgé de 54 ans, opéré de décollement de rétine de l'œil droit par vitrectomie associée à une cryopexie de la déhiscence rétinienne et un tamponnement par hexafluorure de soufre (SF6), et chez qui l'examen à j1 du post opératoire trouve un aspect de cataracte sous-capsulaire postérieure en « plumes de paon ».

**Figure 1:**
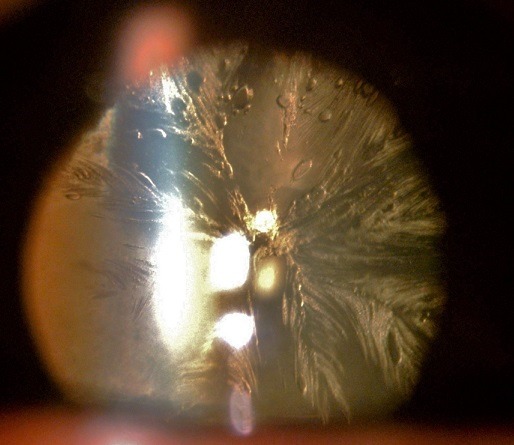
Aspect de cataracte de l’oeil droit en « plumes de paon »

